# Effect of RECK Gene Polymorphisms on Hepatocellular Carcinoma Susceptibility and Clinicopathologic Features

**DOI:** 10.1371/journal.pone.0033517

**Published:** 2012-03-12

**Authors:** Tsung-Te Chung, Chao-Bin Yeh, Yi-Ching Li, Shih-Chi Su, Ming-Hsien Chien, Shun-Fa Yang, Yi-Hsien Hsieh

**Affiliations:** 1 Institute of Medicine, Chung Shan Medical University, Taichung, Taiwan; 2 Department of Otolaryngology, Show Chwan Memorial Hospital, Changhua, Taiwan; 3 Department of Emergency Medicine, School of Medicine, Chung Shan Medical University, Taichung, Taiwan; 4 Department of Emergency Medicine, Chung Shan Medical University Hospital, Taichung, Taiwan; 5 Department of Pharmacology, Chung Shan Medical University, Taichung, Taiwan; 6 Department of Urology, The University of Texas MD Anderson Cancer Center, Houston, Texas, United States of America; 7 Taipei Medical University-Wan Fang Hospital, Taipei, Taiwan; 8 Graduate Institute of Clinical Medicine, College of Medicine, Taipei Medical University, Taipei, Taiwan; 9 Department of Biochemistry, School of Medicine, Chung Shan Medical University Hospital, Taichung, Taiwan; 10 Institute of Biochemistry and Biotechnology, Chung Shan Medical University, Taichung, Taiwan; 11 Clinical Laboratory, Chung Shan Medical University Hospital, Taichung, Taiwan; University of Navarra, Spain

## Abstract

**Background:**

The reversion-inducing-cysteine-rich protein with Kazal motifs (RECK) down-regulation has been confirmed in numerous human cancers and is clinically associated with metastasis. This study investigates the potential associations of RECK single-nucleotide polymorphisms (SNPs) with hepatocellular carcinoma (HCC) susceptibility and its clinicopathologic characteristics.

**Methodology/Principal Findings:**

A total of 135 HCC cancer patients and 501 cancer-free controls were analyzed for four RECK SNPs (rs10814325, rs16932912, rs11788747, and rs10972727) using real-time PCR and PCR-RFLP genotyping analysis. After adjusting for other co-variants, the individuals carrying RECK promoter rs10814325 inheriting at least one C allele had a 1.85-fold [95% confidence interval (CI), 1.03–3.36] risk of developing HCC compared to TT wild type carriers. The HCC patients, who carried rs11788747 with at least one G allele, had a higher distant metastasis risk than wild type probands.

**Conclusions:**

RECK gene polymorphisms might be a risk factor increasing HCC susceptibility and distant metastasis in Taiwan.

## Introduction

Hepatocellular carcinoma (HCC) is the sixth most common cancer worldwide, with 750 000 newly diagnosed cases in 2008. HCC is the third most common cause of cancer mortality [1]. Taiwan has the third highest incidence area in the world, with a 35.7/10^5^ age-standardized rate in 2008. HCC was the leading cause of cancer death among men, with a mortality of 39.3/10^5^, and the second among women, with mortality of 14.7/10^5^ in 2008 [2]. The development of HCC is a multistep and complex process. Multiple environmental risk factors, including chronic hepatitis B virus (HBV) or hepatitis C virus (HCV) infection, cirrhosis, carcinogen exposure, and a variety of genetic factors contribute to hepatocarcinogenesis [3], [4]. Cumulative studies have suggested the associations between HCC cancer risk and SNPs in selected candidate genes. For example, insulin-like growth factor (IGF)-2, IGF-2R, plasminogen activator inhibitor (PAI)-1, and matrix metalloproteinase (MMP) 14 [5]–[7] are HCC risk factors.

The reversion-inducing-cysteine-rich protein with Kazal motifs (RECK) gene is a novel transformation suppressor gene against activated *ras* oncogenes, and induces flat reversion in v-K-ras-transformed NIH/3T3 cells [8], [9]. The RECK gene encodes a membrane-anchored glycoprotein that can negatively regulate matrix metalloproteinases (MMPs) and inhibit tumor invasion, angiogenesis, and metastasis. RECK-expressing tumors show a significant reduction in microvessel density and branching, and result in tumor tissue death in mice [8], [10]. RECK down-regulation or promoter hypermethylation have been confirmed in many human cancers, including pancreatic cancer, breast cancer, lung cancer, colorectal cancer, cholangiocarcinoma, gastric cancer, prostate cancer, oral cancer, esophageal cancer, and osteosarcoma, and correlated with tumor metastasis or poor prognosis [11]–[23].

**Figure 1:**
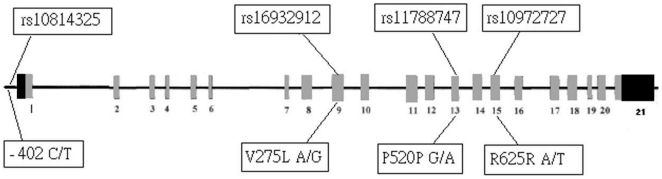
The location of human RECK gene single-nucleotide polymorphisms (SNPs) and functional amino acid.

A previous study showed high RECK mRNA expression in HCC tumor tissues from patients with better survival and less invasive clinicopathologic features [24]. However, to our knowledge, no study has investigated the role of RECK gene variants in HCC development and clinical factors. This study performs a case-control study for four SNPs located in the promoter or exon regions of the RECK gene (Fig. 1) to analyze the associations between RECK gene SNPs and HCC susceptibility and clinicopathologic characteristics

## Materials and Methods

### Study subjects and specimen collection

The present hospital based case-control study recruited 135 HCC patients between 2007 to 2010 in Chung Shan Medical University Hospital, Taiwan. The diagnosis of HCC was according to the characteristic criteria of the national guidelines for HCC, such as liver tumor tissue diagnosed by histology or cytology irrespective of α-fetoprotein (AFP) titer where imaging data, either computed tomography or magnetic resonance imaging, showed one of following three cases: (1) One or more liver masses ≧2 cm in diameter; (2) One imaging data with early enhancement and a high level of AFP ≧400 ng/mL; (3) One imaging data with early arterial phase contrast enhancement plus early venous phase contrast washout. Meanwhile, 501 race- and ethnic group-matched non-cancer evidence community residents who entered the hospital for health check-ups were enrolled as the control group.

HCC patients were staged clinically at the time of diagnosis according to the TNM staging system of the American Joint Committee on Cancer (AJCC) (2002) [25]. Liver cirrhosis was diagnosed with liver biopsy, abdominal sonography, or biochemical evidence of liver parenchymal damage with endoscopic esophageal or gastric varices. The patients' clinicopathological characteristics, such as clinical staging, tumor size, lymph node metastasis, distant metastasis, hepatitis B surface antigen (HBsAg), antibody to HCV (anti-HCV), liver cirrhosis, AFP, aspartate aminotransferase (AST) and alanine aminotransferase (ALT), were verified by chart review. The whole blood specimens collected from healthy controls and HCC patients, were placed in tubes containing ethylenediaminetetraacetic acid (EDTA) and immediately centrifuged and stored at −80°C. Before the conduction of this study, approval from the Institutional Review Board of Chung Shan Medical University Hospital was obtained, and informed written consent was obtained from each individual.

### Genomic DNA extraction

Genomic DNA was extracted by QIAamp DNA blood mini kits (Qiagen, Valencia, CA) according to the instructions of manufacturer. DNA was dissolved in TE buffer [10 mM Tris (pH 7.8), 1 mM EDTA] and then quantified by a measurement of OD260. Final preparation was stored at −20°C and used as templates for polymerase chain reaction (PCR).

### Polymerase chain reaction-restriction fragment length polymorphism

The RECK rs16932912, rs11788747 and rs10972727 gene polymorphisms were determined by polymerase chain reaction-restriction fragment length polymorphism (PCR-RFLP) assay as previously described [21]. The primer sequences and restriction enzyme for analysis of the RECK gene polymorphisms are described in Table 1. The PCR was performed in a 10 µL volume containing 100 ng DNA template, 1.0 µL of 10× PCR buffer (Invitrogen, Carlsbad, CA), 0.25 U of Taq DNA polymerase (Invitrogen, Carlsbad, CA), 0.2 mM dNTPs (Promega, Madison, WI), and 200 nM of each primer (MDBio Inc, Taipei, Taiwan). The PCR cycling conditions were 5 min at 94°C followed by 35 cycles of 1 min at 94°C, 1 min at 60°C, and 2 min at 72°C, with a final step at 72°C for 20 min. Ten µl aliquot of PCR product was subjected to digestion at 37°C for 4 h in a 15 µL reaction buffer containing 1.5 µL 10× buffer (New England Biolabs) and 5 U of *Tfi*I, *Rsa*I and *HpyCH4*IV (New England Biolabs, Beverly, MA) for RECK rs16932912, rs11788747 and rs10972727, respectively. To validate results from PCR-RFLP, around 10% of assays were repeated and several cases of each genotype were confirmed by the DNA sequence analysis.

**Table 1:** Primer sequences and PCR-RFLP conditions for amplification of *RECK* SNPs.

SNP	Sequences	Product	Enzyme
RECKrs16932912	5′-TGGAGATTGTTGATGGTCTC-3 5′-CGGTACACAATGCTCAATAC-3′	G/G: 353 bpA/A: 250 bp, 103 bp	*Tfi*I
RECKrs11788747	5′-GTAGAAGAAGTGACTCATCC-3 5′-ATCTCACTCCGAAGATAACC-3′	A/A: 242 bpG/G: 140 bp, 102 bp	*Rsa*I
RECKrs10972727	5′-TTCTGTCAGGTCATGGAACA-3′ 5′-TGCAGTTAAGACTGGAGAAG-3′	T/T: 224 bpA/A: 119 bp, 105 bp	*HpyCH4*IV

### Real-time PCR

The allelic discrimination of the RECK rs10814325 gene polymorphisms was assessed with the ABI StepOne™ Real-Time PCR System (Applied Biosystems) and analyzed using SDS v3.0 software (Applied Biosystems), using the TaqMan assay (assay IDs: C_27084758_10). The final volume for each reaction was 5 µL, containing 2.5 µL TaqMan Genotyping Master Mix, 0.125 µL TaqMan probes mix, and 10 ng genomic DNA. The real-time PCR reaction included an initial denaturation step at 95°C for 10 min, followed by 40 cycles, each consisting of 95°C for 15 sec and 60°C for 1 min.

### Statistical analysis

Hardy–Weinberg equilibrium was assessed using a chi-square goodness-of-fit test for biallelic markers. A Mann–Whitney U-test and a Fisher's exact test were used to compare the differences of age as well as demographic characteristics distributions between controls and HCC patients. The adjusted odds ratio (AOR) with their 95% confidence intervals (CIs) of the association between genotype frequencies and HCC risk as well as clinical pathological characteristics were estimated by multiple logistic regression models after controlling for other covariates, A *p* value<0.05 was considered significant. Rs10814325 SNP was predicted to change transcription factor binding sites by TRANSFAC (URL: http://thr.cit.nih.gov/molbio/signal/) [12]. The data were analyzed on SAS statistical software (Version 9.1, 2005; SAS Institute Inc., Cary, NC).

## Results

This study analyzes the demographic characteristics of sample specimens and found that 41.9% (210 of 501) and 34.8% (47 of 135) of healthy control subjects and patients with HCC, respectively, who had consumed alcohol; 41.1% (206 of 501) and 40.7% (55 of 135) of healthy controls and patients with HCC, respectively, had smoked. The distributional differences of alcohol (*p* = .136) and tobacco consumption (*p* = .937) between healthy controls and patients with HCC were insignificant, whereas age distribution (control: 52.9±14.48; HCC: 64.24±11.08) (*p*<.001) and gender distribution (*p*<.001) between the 2 subgroups were significantly different (Table 2). To reduce the possible interference of the confounding variables, we used adjusted odds ratios (AORs) with 95% confidence intervals (CIs) were estimated by multiple logistic regression models after controlling for age and gender in each comparison. Table 3 shows the genotype distributions and the association between HCC and gene polymorphisms of RECK. The alleles with the highest distribution frequency for rs10814325, rs16932912, rs11788747, and rs10972727 genes of RECK in both recruited HCC patients and healthy controls were heterozygous T/C, homozygous G/G, homozygous A/A, and homozygous T/T, respectively. After adjusting variables, people with rs10814325 TC, CC, TC+CC showed a 2.112-fold (95% CI: 1.108–4.028), 2.184-fold (95%CI: 1.211–3.939), or 1.85-fold (95%CI: 1.027–3.361) higher risk of HCC compared to wild type individuals. People with rs16932912, rs11788747, and rs10972727 genes of RECK polymorphism showed no higher risk of HCC compared to wild type individuals.

**Table 2:** The distributions of demographical characteristics in 501 healthy controls and 135 patients with HCC.

Variable	Controls (N = 501)	Patients (N = 135)	*p* value
**Age (yrs)**	**Mean ± S.D.**	**Mean ± S.D.**	
	52.9±14.48	64.24±11.08	*P*<0.001*
**Gender**	**n (%)**	**n (%)**	
Male	415 (82.8%)	92 (68.1%)	
Female	86 (17.2%)	43 (31.9%)	*p*<0.001*
**Alcohol consumption**			
No	291 (58.1%)	88 (65.2%)	
Yes	210 (41.9%)	47 (34.8%)	*p* = 0.136
**Tobacco consumption**			
No	295 (58.9%)	80 (59.3%)	
Yes	206 (41.1%)	55 (40.7%)	*p* = 0.937

Mann-Whitney U test or Fisher's exact test was used between healthy controls and patients with HCC.

* , *p* value<0.05 as statistically significant.

**Table 3:** Adjusted odds ratio (AOR) and 95% confidence interval (CI) of HCC associated with *RECK* genotypic frequencies.

Variable	Controls (N = 501) n (%)	Patients (N = 135) n (%)	OR (95% CI)	AOR (95% CI)
**rs10814325**				
TT	143 (28.5%)	21 (15.6%)	1.00	1.00
TC	225 (44.9%)	72 (53.3%)	**2.179 (1.283–3.700)**	**2.11 (1.11–4.03)**
CC	133 (26.5%)	42 (31.1%)	**2.150 (1.211–3.820)**	**2.18 (1.21–3.94)**
TC+CC	358 (71.5%)	114 (84.4%)	**2.168 (1.310–3.590)**	**1.85 (1.03–3.36)**
**rs16932912**				
GG	272 (54.3%)	64 (47.4%)	1.00	1.00
GA	192 (38.3%)	57 (42.2%)	1.262 (0.844–1.886)	1.41 (0.88–2.27)
AA	37 (7.4%)	14 (10.4%)	1.608 (0.821–3.151)	1.81 (0.75–4.37)
GA+AA	229 (45.7%)	71 (52.6%)	1.318 (0.900–1.928)	1.48 (0.94–2.33)
**rs11788747**				
AA	281 (56.1%)	83 (61.5%)	1.00	1.00
AG	192 (38.3%)	41 (30.4%)	0.723 (0.477–1.097)	0.66 (0.40–1.07)
GG	28 (5.6%)	11 (8.1%)	1.330 (0.635–2.785)	1.27 (0.51–3.16)
AG+GG	220 (43.9%)	52 (38.5%)	0.800 (0.542–1.181)	0.74 (0.47–1.17)
**rs10972727**				
TT	279 (55.7%)	76 (56.3%)	1.00	1.00
TA	191 (38.1%)	54 (40.0%)	1.038 (0.700–1.540)	1.03 (0.64–1.64)
AA	31 (6.2%)	5 (3.7%)	0.592 (0.223–1.575)	0.48 (0.16–1.46)
TA+AA	222 (44.3%)	59 (43.7%)	0.976 (0.665–1.431)	0.96 (0.61–1.51)

The odds ratios (ORs) with their 95% confidence intervals were estimated by logistic regression models.

The adjusted odds ratios (AORs) with their 95% confidence intervals were estimated by multiple logistic regression models after controlling for age, gender, alcohol and tobacco consumption.

The distribution frequency of clinical statuses and RECK genotypes frequencies in HCC patients were estimated to clarify the role of RECK gene polymorphisms in the clinicopathologic states of HCC patients, including TNM clinical staging, primary tumor size, lymph node involvement, distant metastasis, hepatitis B surface antigen (HBsAg), antibody to HCV (anti-HCV), and liver cirrhosis. There was no observed significant association between the rs10814325, rs16932912, and rs10972727 gene polymorphisms and clinicopathologic states. However, among HCC patients, those who had rs11788747 SNPs also had a higher risk of distant metastasis (*p* = .003) than wild type patients (Table 4), but no difference existed in HBsAg, anti-HCV, Child-Pugh grade or liver cirrhosis (Table 5).

**Table 4:** Clinical TNM staging and *RECK* genotypic frequencies in 135 HCC patients.

Variable	rs10814325			rs16932912			rs11788747			rs10972727		
	TT (N = 21)	TC+CC (N = 144)	*p* value	GG (N = 64)	GA+AA (N = 71)	*p* value	AA (N = 83)	AG+GG (N = 52)	*p* value	TT (N = 76)	TA+AA (N = 59)	*p* value
	n (%)	n (%)		n (%)	n (%)		n (%)	n (%)		n (%)	n (%)	
**Clinical stage**												
Stage I/II	12 (57.1%)	72 (63.2%)	0.601	38 (59.4%)	46 (64.8%)	0.517	54 (65.1%)	30 (57.7%)	0.390	49 (64.5%)	35 (59.3%)	0.540
Stage III/IV	9 (49.2%)	42 (36.8%)		26 (40.6%)	25 (35.2%)		29 (34.9%)	22 (42.3%)		27 (35.5%)	24 (40.7%)	
**Tumor size**												
≦T2	13 (61.9%)	72 (64.9%)	0.791	41 (64.1%)	46 (64.8%)	0.930	55 (66.3%)	32 (61.5%)	0.577	51 (67.1%)	36 (61.0%)	0.464
>T2	8 (38.1%)	42 (35.1%)		23 (35.9%)	25 (35.2%)		28 (33.7%)	20 (38.5%)		25 (32.9%)	23 (39.0%)	
**Lymph node metastasis**												
No	19 (90.5%)	109 (95.6%)	0.298	60 (93.8%)	68 (95.8%)	0.442	79 (95.2%)	49 (94.2%)	0.550	72 (94.7%)	56 (94.9%)	0.639
Yes	2 (9.5%)	5 (4.4%)		4 (6.3%)	3 (4.2%)		4 (4.8%)	3 (5.8%)		4 (5.3%)	3 (5.1%)	
**Distant metastasis**												
No	19 (90.5%)	110 (96.5%)	0.235	59 (92.2%)	70 (98.6%)	0.082	83 (100.0%)	46 (88.5%)	0.003*	75 (98.7%)	54 (91.5%)	0.057
Yes	2 (9.5%)	4 (3.5%)		5 (7.8%)	1 (1.4%)		0 (0%)	6 (11.5%)		1 (1.3%)	5 (8.5%)	

* *p* value<0.05.

**Table 5:** Clinical status and *RECK* genotypic frequencies in 135 HCC patients.

Variable	rs10814325			rs16932912			rs11788747			rs10972727		
	TT (N = 21)	TC+CC (N = 144)	*p* value	GG (N = 64)	GA+AA (N = 71)	*p* value	AA (N = 83)	AG+GG (N = 52)	*p* value	TT (N = 76)	TA+AA (N = 59)	*p* value
	n (%)	n (%)		n (%)	n (%)		n (%)	n (%)		n (%)	n (%)	
**HBsAg**												
Negative	10 (47.6%)	71 (62.3%)	0.208	37 (57.8%)	44 (62.0%)	0.622	51 (61.4%)	30 (57.7%)	0.665	45 (59.2%)	36 (61.0%)	0.832
Positive	11 (52.4%)	43 (37.7%)		27 (42.2%)	27 (38.0%)		32 (38.6%)	22 (42.3%)		31 (40.8%)	23 (39.0%)	
**Anti-HCV**												
Negative	8 (38.1%)	55 (48.2%)	0.392	31 (48.4%)	32 (45.1%)	0.695	41 (49.4%)	22 (42.3%)	0.422	41 (53.9%)	22 (37.3%)	0.054
Positive	13 (61.9%)	59 (51.8%)		33 (51.6%)	39 (54.9%)		42 (50.6%)	30 (57.7%)		35 (46.1%)	37 (62.7%)	
**Child-Pugh grade**												
A	15 (71.4%)	84 (73.7%)	0.830	49 (76.6%)	50 (70.4%)	0.421	61 (73.5%)	38 (73.1%)	0.957	56 (73.7%)	43 (72.9%)	0.917
B or C	6 (28.6%)	30 (26.3%)		15 (23.4%)	21 (29.6%)		22 (26.5%)	14 (26.9%)		20 (26.3%)	16 (27.1%)	
**Liver cirrhosis**												
Negative	5 (23.8%)	30 (26.3%)	0.810	17 (26.6%)	18 (25.4%)	0.873	23 (27.7%)	12 (23.1%)	0.550	21 (27.6%)	14 (23.7%)	0.608
Positive	16 (76.2%)	84 (73.7%)		47 (73.4%)	53 (74.6%)		60 (72.3%)	40 (76.9%)		55 (72.4%)	45 (76.3%)	

AFP, AST, and ALT are common clinical pathological markers of HCC. To clarify the relationship between the progress of clinical status and the level of clinical pathological markers in HCC patients, this study also analyzes the levels of these pathological markers associated with RECK genotypic frequencies. Table 6 shows that the levels of ALT were significantly different between the rs11788747 AA and AG/GG genotypes (*p*<.05).

**Table 6:** Association of *RECK* genotypic frequencies with HCC laboratory status.

Characteristic	α-Fetoprotein^a^(ng/mL)	AST^a^(IU/L)	ALT^a^(IU/L)	AST/ALT ratio^a^
**rs10814325**				
TT	165.8±50.6	191.4±70.3	128.5±24.5	1.34±0.18
TC/CC	3999.3±1542.3	178.7±33.6	144.1±34.4	1.53±0.11
*p* value	0.289	0.880	0.980	0.465
**rs16932912**				
GG	3582.8±2169.5	155.4±30.7	136.1±23.0	1.35±0.10
GA/AA	3240.8±1548.8	203.4±50.6	148.9±37.7	1.64±0.16
*p* value	0.897	0.431	0.777	0.125
**rs11788747**				
AA	3261.3±1376.1	220.9±46.7	179.4±35.2	1.47±0.10
AG/GG	3629.1±2606.0	116.4±46.7	84.6±13.2	1.56±0.19
*p* value	0.892	0.093	0.040*	0.659
**rs10972727**				
TT	3317.6±1491.4	225.2±50.7	182.1±42.5	1.51±0.12
TA/AA	3512.9±2307.8	122.2±21.8	95.5±13.2	1.49±0.16
*p* value	0.941	0.095	0.052	0.919

Mann-Whitney U test was used between two groups.

a Mean ± S.E.

* *p* value<0.05.

## Discussion

This study provides novel information on the effects of single-nucleotide polymorphisms of RECK on HCC susceptibility and clinicopathologic status association. The genomic structure for the RECK gene has been identified on the chromosome region 9p13→p12. The RECK gene includes 21 exons and 20 introns, and 13 SNPs were identified [26]. Among 13 SNPs, rs16932912, rs11788747, and rs10972727 were found within the coding sequence in exons 9, 13, and 15, respectively [26]. These polymorphisms are non-synonymous and changed amino acid sequence as well as RECK protein structure. For example, Valine changes to Lysine in rs16932912 SNP. Rs10814325 SNP was located at promoter sites and was predicted to change transcription factor binding sites by TRANSFAC [12]. Although the functional importance of rs10814325 SNPs has not been tested experimentally, clinical research from Germany shows that breast cancer patients carrying rs10814325 -402 T/C SNP have a greater chance of survival than those carrying T/T wild type [12]. In the present study, individuals carrying the RECK promoter rs10814325 inheriting at least one C allele had a 1.85-fold higher risk of HCC compared to TT wild type carriers. However, no significant association was present between the rs10814325 gene polymorphisms and clinicopathologic states. The data are apparently contradictory in relation to carcinogenesis and probably survival. However, the potential mechanism and functional consequences of the promoter sequence change should be elucidated in the laboratory and clinically as well. Meanwhile, in this study, the frequencies of rs10814325 T/T, T/C and C/C were 28.5%, 44.9% and 26.5%, respectively. However, in Sweden, the genotypes distribution of RECK rs10814325 T/T, T/C and C/C were 83.7%, 15.8% and 0.5%, respectively [12]. Obviously, there is an ethnicity-related variation in the frequency of rs10814325 polymorphisms. The contradictory findings of previous research may be the result of the different ethnic backgrounds of patients.

This study shows that individuals carrying the RECK promoter rs10814325 polymorphism have a 1.85-fold risk of developing HCC compared to wild type carriers. In an animal model, the administration of black-tea polyphenon-B significantly reduces the incidence of dimethylaminoazobenzene (DAB)-induced hepatomas as evidenced by modulation of RECK, matrix metalloproteinase (MMP)-2, MMP-9, and the tissue inhibitor of matrix metalloproteinase-2 [27]. The alternation of RECK promoter affinity and RECK expression is likely the result of activating the extracellular signal-regulated kinase (ERK) pathway [28], [29]. In a human model, up-regulation of the mitogen-activated protein kinase (MAPK) pathway and genes is associated with an activated cell cycle and may be of critical importance to the formation and maintenance of hepatocellular carcinoma [30], [31]. These findings might be the molecular hepatocarcinogenesis of RECK, but the mechanism should be elucidated in laboratory and clinical settings.

This study shows that HCC patients carrying rs11788747 polymorphisms had a higher risk of distant metastasis (*p* = .003) than wild type carriers. Previous research confirms the relationships of RECK expression and gene status with tumor metastasis. Using Matrigel invasion chamber assay in vitro and in experimental and spontaneous metastasis assays in vivo, the experiments in this study artificially restored RECK expression in tumor cells (such as HT1080 fibrosarcoma or B16 melanoma), in which endogenous RECK was undetectable. This approach greatly suppresses their invasive and metastatic potentials [8]. Low RECK expression colorectal cancer and esophageal cancer patients exhibited more lymph node metastasis [14], [32]. In non-small cell lung cancer patients, RECK promoter methylation has a higher incidence of lymph node metastasis [33]. In betel quid-chewing oral cancer patients, those who have the RECK rs10814325 polymorphism have a higher risk of neck lymph node metastasis than wild type carriers [21].

This is the first study to associate the RECK gene polymorphisms with risk of HCC. Results showed RECK promoter SNP individuals increase HCC susceptibility, and rs11788747 polymorphism HCC patients had a higher risk of distant metastasis. These findings suggest that RECK plays a role in HCC carcinogenesis and metastasis. However, the number of case and control people in this study was relatively small. Additional studies with larger sample sizes are needed to validate the genetic effects of the RECK polymorphisms on HCC. Further collection of samples should investigate the RECK sequence variants and their biological function in Taiwanese HCC patients.
